# Current practice of neonatal resuscitation documentation in North America: a multi-center retrospective chart review

**DOI:** 10.1186/s12887-015-0503-8

**Published:** 2015-11-14

**Authors:** Matthew S. Braga, Prakash Kabbur, Pradeep Alur, Michael H. Goodstein, Kari D. Roberts, Katie Satrom, Sandesh Shivananda, Ipsita Goswami, Mariann Pappagallo, Carrie-Ellen Briere, Gautham Suresh

**Affiliations:** Geisel School of Medicine at Dartmouth, The Dartmouth Institute for Health Policy and Clinical Practice, Children’s Hospital at Dartmouth, One Medical Center Drive, Lebanon, NH 03756 USA; Kapi’olani Medical Center for Women and Children, Neonatology, 1319 Punahou St, Honolulu, HI 96826 USA; Wellspan Health, York Hospital, Neonatology, 1001 S. George St., York, 17403 NY USA; University of Minnesota Masonic Children’s Hospital, Neonatology, 2450 Riverside Ave, Minneapolis, MN 55454 USA; McMaster University, McMaster Children’s Hospital, Neonatology, 1200 Main St W, Hamilton, ON L8N 3Z5 Canada; University of Connecticut Health Center, Connecticut Children’s Medical Center, Neonatology, 282 Washington St., Farmington, 06106 CT USA; Texas Children’s Hospital, Baylor College of Medicine, Neonatology, 6621 Fannin St, Houston, TX 77030 USA

**Keywords:** Neonatal resuscitation, Neonatal documentation, Neonatal resuscitation program, Code documentation, Documentation

## Abstract

**Background:**

To determine the comprehensiveness of neonatal resuscitation documentation and to determine the association of various patient, provider and institutional factors with completeness of neonatal documentation.

**Methods:**

Multi-center retrospective chart review of a sequential sample of very low birth weight infants born in 2013. The description of resuscitation in each infant’s record was evaluated for the presence of 29 Resuscitation Data Items and assigned a Number of items documented per record. Covariates associated with this Assessment were identified.

**Results:**

Charts of 263 infants were reviewed. The mean gestational age was 28.4 weeks, and the mean birth weight 1050 g. Of the infants, 69 % were singletons, and 74 % were delivered by Cesarean section. A mean of 13.2 (SD 3.5) of the 29 Resuscitation Data Items were registered for each birth. Items most frequently present were; review of obstetric history (98 %), Apgar scores (96 %), oxygen use (77 %), suctioning (71 %), and stimulation (62 %). In our model adjusted for measured covariates, the institution was significantly associated with documentation.

**Conclusions:**

Neonatal resuscitation documentation is not standardized and has significant variation. Variation in documentation was mostly dependent on institutional factors, not infant or provider characteristics. Understanding this variation may lead to efforts to standardize documentation of neonatal resuscitation.

## Background

The quality of resuscitation and stabilization of a neonate immediately after birth has a significant effect on neonatal morbidity and mortality, particularly in high-risk infants such as very-low-birth-weight infants [1]. In order to ensure optimal care immediately after birth, the quality of such resuscitation should be monitored within an institution and across institutions. The most practical source of data to evaluate neonatal resuscitation performance is the documentation by health professionals in the medical chart about the baby’s condition and the care provided during resuscitation. Several reports of the quality of medical documentation suggest room for improvement [2–7]. A recent single-center study from Sweden reported that in 45 % of cases, documentation of the neonatal resuscitation was incomplete [8]. The 2000 International Liaison Committee on Resuscitation (ILCOR) for Neonatal Resuscitation recommendations on documentation state: “Assign Apgar scores at 1 and 5 min after birth and then sequentially every 5 min until vital signs have stabilized. Complete documentation must also include a narrative description of interventions performed and their timing” [9]. The updated 2010 ILCOR guidelines do not include any specific recommendations for neonatal resuscitation documentation [10, 11].

There have been no studies so far about how well neonatal resuscitation is documented in North America and what deficiencies exist in such documentation. We hypothesized that documentation of neonatal resuscitation frequently lacks inclusion of key items. Therefore we conducted this study in order to: (1) Develop a comprehensive set of items that should ideally be included in resuscitation documentation, (2) Assess the frequency, and nature of included, missing, and incomplete documentation in the medical records of high-risk neonates in multiple institutions, (3) Identify factors associated with completeness of documentation.

## Methods

This was a retrospective multicenter study conducted in 6 North American institutions. Institutional Review Board approval and waiver of consent was obtained at each participating institutions including; Dartmouth College, University of Minnesota, McMaster University, University of Connecticut, York Hospital WellSpan Health, Hawaii Pacific Health.

Using a modified Delphi process [12] with a panel of 10 neonatologists from the participating institutions, we developed a comprehensive list of items that could possibly be included in neonatal resuscitation documentation. The panel was instructed to develop a comprehensive list of items that was all-inclusive, and that could serve as a precursor to a short practical list of items to routinely monitor the performance and quality of neonatal resuscitation. Two investigators (MB and GS) developed the initial list of items and circulated it to the expert panel, which reviewed the list in an iterative fashion to generate a final comprehensive list of 29 Resuscitation Data Items. We also developed operational definitions for each of these items. Two investigators (MB and GS) then abstracted the Resuscitation Data Items from 5 records in their institution to pilot test and refine the operational definitions.

We then sought to review the actual neonatal resuscitation documentation in the charts of 50 inborn very low birth weight (VLBW, ≤1500 g) infants in each institution that were born sequentially in 2013. We chose VLBW infants because they are readily identifiable and have a high likelihood of resuscitation interventions such as intubation, immediately after delivery. This ensured that we had an adequate number of relevant resuscitation events to evaluate during chart review. We excluded infants ≥1500 g, infants <1500 g with absent or unavailable medical records, and out-born infants. If an institution could not locate a medical record for an infant, we used the next available sequential medical record of an eligible infant.

The documentation reviewed included all patient notes written by physicians, nurse practitioners or physician assistants for the first two calendar days of life for these infants. These notes contained resuscitation records (including ‘code sheets’), initial history and physical documentation, procedure notes, and progress/event notes for each infant. We did not review nursing or respiratory therapy flow-sheets or medication administration records as we decided that ideally relevant information related to resuscitation of the infant should be included in one location such as the delivery note. Maternal records were not reviewed. Participating institutions sent de-identified copies of these records to the principal investigator’s institution by secure mail for review and data extraction. The principal investigator (MB) reviewed the charts, and extracted data. Study data were collected and managed using REDCap (Research Electronic Data Capture) electronic data capture tools hosted at Dartmouth. REDCap is a secure, web-based application designed to support data capture for research studies [13]. Investigators from one center (Institution E on Tables and Figures) performed the chart review themselves. Investigators at this center received detailed instructions and definitions for each item, and performed pilot data abstraction of five records prior to reviewing the 50 definitive charts from their institution. Records from the principal investigator’s institution were printed from the electronic medical record and reviewed locally for data extraction.

To identify hospital-level variables we obtained characteristics of each neonatal intensive care unit from the investigators.

Number of items documented per record: In each infant’s record we assessed whether or not each Resuscitation Data Item was documented. An item was counted as ‘documented’ if the notes reviewed mentioned that item in any form. This could include a description of an intervention (such as bag-mask-ventilation) being provided to the infant, or a statement that such an item was not provided to the infant, or was not required by the infant. An item was counted as ‘not documented’ if there was no mention at all in the reviewed notes of the item. The maximum possible number of documented items in the record was 29.

### Analysis

#### Institutional characteristics and patient demographics

We derived descriptive statistics for the characteristics of participating institutions, and the demographic characteristics of patients whose charts were reviewed, including: birth weight and gestational age, multiple gestation, method of delivery, and presence of congenital anomalies. Patient characteristics were analyzed for the overall sample as well as for each institution.

#### Descriptive analysis of documentation

The number and proportion of missing items in resuscitation documentation was calculated for the overall sample and for each institution.

#### Identification of factors associated with deficient documentation

Covariates associated with documentation, such as the professional role of the person documenting the resuscitation (attending physician, fellow, resident, nurse practitioner, physician’s assistant), time of delivery, location of delivery, and use of a note template were identified through univariate analysis and multivariate analysis. If a note was written first by one professional and then another provider added to it (for example if an attending physician wrote an addendum to a resident’s note), the documentation was attributed to the primary author of the note. The univariate analysis sought factors that were significantly associated with the number of items documented per record, and was followed by a multivariate analysis to derive estimates of strength of association adjusted for confounding. Specifically we used a multiple linear regression model with independent variables (covariates) entered into the model. Covariates in our final model included the institution, primary documenter, gestational age, birth weight, multiparity, and delivery method.

All statistics were calculated using STATA 13.1.

## Results

The characteristics of the six participating institutions are depicted in Table 1. The number of beds per Neonatal Intensive Care Unit ranged from 30 to 70, the number of attending physicians ranged from 5 to 15, the total deliveries per year ranged from 644 to 3100, and VLBW deliveries per year ranged from 55 to 250. Most had pediatric residents and NICU fellows and all had advanced practice nurses (nurse practitioners).

**Table 1:** Characteristics of participating institutions

Institution	A	B	C	D	E	F
Number of NICU^a^ Beds	30	40	70	38	47	49
Number of NICU^a^ Attendings	5	15	14	6	12	10
Number of Deliveries per year	1100	644	7000	3100	3000	2500
Number of Very Low Birth Weight Deliveries per year	69	55	150	75	250	130
Stand Alone NICU^a^ Resuscitation Room	Yes	Yes	No	No	Yes	No
In-House Attending Coverage	No	No	Yes	Yes	No	No
Pediatric Residency Program	Yes	Yes	Yes	No	Yes	Yes
Fellowship Program	Yes	Yes	Yes	No	Yes	Yes
Advance Practice Nurse Practitioners	Yes	Yes	Yes	Yes	Yes	Yes
Institutional Guidelines for Neonatal Resuscitation Documentation	No	Yes	No	Yes	Yes	No
Number of Records Reviewed	50	24	39	50	50	50

^a^NICU-Neonatal Intensive Care Unit

We reviewed 263 records from these six institutions. Although we aimed to review 50 records from each institution, 26 records were not available from one institution, and 11 records from another institution were excluded as they belonged to out-born infants. One institution was only able to contribute 24 records due to research personnel issues and a few records from other institutions were excluded due to not fully meeting inclusion criteria (out-born infants, for example). The characteristics of the infants whose charts were reviewed are depicted in Table 2.

**Table 2:** Characteristics of very low birth weight infants

Characteristics	Summary Measure (*n* = 263)
Demographics	
Mean Birth Weight (SD) in grams	1050 (315)
Mean Gestational Age (SD) in weeks	28.4 (2.8)
Singleton Gestation	69.2 %
Twin Gestation	24.0 %
Triplet Gestation^1^	5.3 %
Vaginal Delivery	25.9 %
Congenital Anomaly documented	7.2 %
Born during day shift (8:01–20:00)	59.6 %
Median 1 min Apgar (IQR)	6 (4, 7)
Median 5 min Apgar (IQR)	8 (7, 9)
Respiratory Support	
Continuous Positive Airway Pressure documented	52.4 %
Bag Mask Ventilation documented	58.6 %
Intubation documented	53.2 %
Surfactant administration documented	39.9 %
Cardiovascular Support	
Umbilical Line placement documented	20.2 %
Chest Compressions documented	4.6 %
Epinephrine administration documented	2.7 %

^1^One Pregnancy was documented as > triplet

The mean (SD) birth weight was 1050 (315) grams, the mean (SD) gestational age was 28.4 (2.8) weeks, 69 % were singleton, 74 % were delivered by Cesarean section, and 60 % were born during the day shift (8 am-8 pm). The median (IQR) Apgar scores at 1 and 5 min were 6 (4, 7) and 8 (7,9) respectively.

**Fig. 1:**
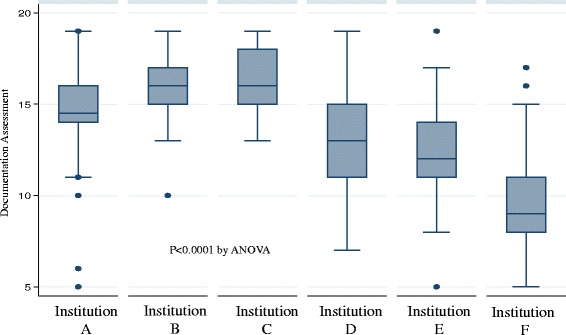
Documentation Assessment for Each Institution. Documentation Score = 1 point for mentioning the following items and 0 points if nothing mentioned; Review of OB history, Briefing done, Equipment check, Personnel Check, Apgar at 1 min, description of APGAR at 1 min, Apgar at 5 min, description of APGAR at 5 min, Delivery Room Temperature, Radiant Warmer, Drying of Baby, Exothermic Mattress, Plastic Wrap, Hat, Temperature Measured in Delivery Room, Pulse Oximetry, Supplemental Oxygen, Airway Clearance/Suctioning, Positioning of Airway, Stimulation, Continuous Positive Airway Pressure, Bag Mask Ventilation, Endotracheal Intubation, Heart Rate with Repeated Assessment and Monitoring, Chest Compressions, Umbilical Line Placement, Assessment Intervention Pattern, Family Presence During/After Resuscitation, Communication with Family During/Immediately Post Resuscitation. Total available points = 29

The mean (SD) number of items documented in a record was 13.2 (3.5). Figure 1 displays the number of items documented at each participating hospital as box and whisker plots.

The percentages of records documenting each of the 29 Resuscitation Data Items for the overall sample are shown in Table 3. For example, only 98 % of all records documented the obstetric history, but only 1 % documented a pre-delivery briefing.

**Table 3:** Resuscitation Data Items with percentage of records that documented each specific item

Data Item	Percent of Records that Documented Specified Data Item
Preparation for Delivery	
Review of OB history	98 %
Briefing Done	1 %
Equipment Check	20 %
Personnel Check	2 %
Apgars	
Apgar at 1 min	96 %
Description of Apgar at 1 min	62 %
Apgar at 5 min	96 %
Description of Apgar at 5 min	62 %
Temperature Management	
Delivery Room Temperature	0 %
Radiant Warmer	60 %
Drying of Baby	48 %
Exothermic Mattress	14 %
Plastic Wrap	30 %
Hat	1 %
Temperature of Baby in Delivery Room	0 %
Pulse Oximetry and Respiratory Support	
Pulse Oximetry	59 %
Supplemental Oxygen	77 %
Airway Clearance/Suctioning	71 %
Positioning of the Airway	3 %
Stimulation	62 %
Continuous Positive Airway Pressure	64 %
Bag Mask Ventilation	68 %
Endotracheal Intubation	65 %
Cardiac Support Provided	
Heart Rate with Repeated Assessment	51 %
Chest Compression	5 %
Umbilical Catheter Placement	30 %
Pattern of Assessment and Intervention Described	66 %
Family Involvement and Interactions	
Family Presence	40 %
Communication with Family	64 %

Records which either mentioned the intervention or mentioned that it was not needed or not done, would be assessed as documented. If a record did not mention an intervention at all, then is was assessed as not documented

Multivariate Linear Regression Model of institutions, providers, and infants and number of items documented per record: In Table 4 we report both the crude regression coefficients for each individual variable with the number of items documented per record, as well as completely adjusted regression coefficients after adjusting for every variable in the table.

**Table 4:** Various characteristics crude and adjusted coefficients on documentation assessment^a^

Characteristic	Crude Coefficient (95 % CI)	Adjusted Coefficient (95 % CI)^b^
Institutional Factors		
Institution		
Institution A	4.6 (3.5, 5.6)^c^	4.4 (3.2, 5.6)^c^
Institution B	6.1 (4.8, 7.4)^c^	6.1 (4.7, 7.6)^c^
Institution C	6.5 (5.4, 7.7)^c^	6.0 (4.7, 7.3)^c^
Institution D	3.1 (2.0, 4.1)	3.7 (2.2, 5.2)^c^
Institution E	2.8 (1.7, 3.9)	2.4 (1.2, 3.6)^c^
Institution F	1.0 (ref.)	1.0 (ref.)
Primary Documenter		
Attending	1.0 (ref.)	1.0 (ref.)
Fellow	1.4 (−0.5, 3.4)	0.9 (−0.4, 2.3)
Resident	1.9 (0.1, 3.7)^c^	1.9 (0.5, 3.2)^c^
Nurse Practitioner	−1.1 (−4.8, 2.5)	0.6 (−0.6, 1.8)
Other	3.8 (1.7, 6.0)^c^	1.4 (−0.4, 3.2)
Infant Factors		
Gestational Age		
< 28 weeks	0.6 (−0.2, 1.5)	0.7 (−0.3, 1.6)
≥ 28 weeks	1.0 (ref.)	1.0 (ref.)
Birth Weight		
< 1000 g	0.6 (−0.2, 1.5)	0.1 (−0.7, 1.0)
≥ 1000 g	1.0 (ref.)	1.0 (ref.)
Birth Time		
8 am-8 pm	1.0 (ref.)	1.0 (ref.)
8 pm-8 am	0.2 (−0.6, 1.1)	0.2 (−0.5, 0.9)
Gestation		
Singleton	1.0 (ref.)	1.0 (ref.)
≥ Twin	−0.6 (−1.5, 0.3)	−0.6 (−1.3, 0.2)
Delivery		
Vaginal	1.0 (ref.)	1.0 (ref.)
Caesarian Section	−0.4 (−1.3, 0.6)	−0.4 (−1.2, 0.4)

^a^Documentation Assessment = see Table 3 for all 29 data elements. Points range from 0–29

^b^Adjusted for all variables in table

^c^Statistically significant characteristics

In our completely adjusted model, only the institution and having a resident document the resuscitation were significantly associated with the number of items documented per record. For example in our completely adjusted model, the number of items documented per record in institution A was greater by 4.56 (95 % CI 3.46, 5.64) than at the reference institution F. In our adjusted model, documentation by a resident was associated with 1.87 (95 % CI 0.52, 3.24) more items documented per record. Removing the institutions that were only able to contribute 24 and 39 records did not meaningfully affect our results. The infant characteristics including; birth weight, birth time, gestational age, presence of congenital anomaly, type of delivery, and multiparity were not significantly associated with the number of items documented per record. Other than having the neonatal resuscitation documented by a resident, the different level of training (attending or fellow) or discipline (nurse practitioner) of the primary documenter were also not significantly associated with the number of items documented per record. We reported our results using a fixed effects model. Analyzing our results with a random methods model did not meaningfully change our results.

Finally, we also analyzed a subset of Resuscitation Data Items, which included only those with <50 % missing documentation. In this analysis the multiple linear regression model did not change meaningfully - significant variation between institutions persisted.

## Discussion

Our study found that the comprehensiveness of documentation of neonatal resuscitation and stabilization of VLBW infants varies significantly across institutions, and that many potentially important items indicative of the quality of the resuscitation were missing from the medical record. This is the first study to examine the comprehensiveness of neonatal resuscitation documentation among multiple institutions, and to identify associated factors. Our findings suggest that there is significant room for improvement in the documentation of neonatal resuscitation and a need for standardization.

We intended to merely describe the comprehensiveness of documentation in the medical record and not its accuracy. Therefore our findings should not be misinterpreted as estimates of the accuracy of resuscitation documentation or the actual appropriateness of the interventions provided. The use of video recordings of neonatal resuscitation has been used successfully at several institutions including ours (MB), and such video recordings would represent the gold standard of documentation and a potential benchmark against which to compare the written documentation [14, 15]. We plan to conduct such a study of documentation accuracy as a follow-up to this comprehensiveness study.

In our multiple linear regression model the number of items documented per record was related to the institution but not to infant characteristics. We attributed the significantly higher number of items documented per record by residents to the fact that their notes often had addenda by attending physicians, thereby increasing the comprehensiveness of documentation. Our analysis suggests the number of items documented per record is institutionally related, which is likely due to policies, use of note templates, and institutional culture around documentation. Therefore we speculate that interventions to educate and facilitate comprehensive documentation may be more effective if applied at an institutional level rather than targeted at individual clinicians.

The strengths of our study include data from a large number of babies from multiple institutions and use of a consecutive sample of VLBW infants at each institution, thereby minimizing selection bias. We also used clear definitions for data extraction and used a comprehensive list of Resuscitation Documentation Items that was developed by experienced neonatologists. Our methods do not imply that all 29 items are essential for inclusion in every resuscitation documentation. This comprehensive list was developed as an initial set of items that could eventually evolve, with expert input and further research, into a short, practical list of core data items for resuscitation. Some of the 29 items could potentially be excluded completely from such a core data set if they are considered to be of trivial importance. Others could be included conditionally – for example, documentation about whether or not chest compressions were performed is unnecessary in a vigorous infant with a strong cry and could be restricted to infants requiring more than the initial steps of resuscitation. Such a core list could then be incorporated into an electronic medical record template (with branching logic guiding the documentation of conditional items such as chest compressions), thereby facilitating quick and essential documentation, and more importantly, quick retrieval of accurate data through field-defined electronic queries. This would make it easy for institutions to generate quality metrics for assessment of how well resuscitation was performed and allow institutional benchmarking.

Our findings should be interpreted in light of certain study limitations. Some of the Resuscitation Data Items may have been documented in parts of the medical record that we did not review (such as the nursing and respiratory flow sheets or maternal records). In addition, unavailable records were excluded and may have biased our results. We restricted our review of the medical record solely to physician and nurse practitioner documentation in the first two days, as we felt that ideally all relevant documentation about resuscitation should be present in one location in the medical record of the infant. We restricted our study to VLBW infants and therefore our findings do not apply to bigger babies. We did not attempt to assign weights to, or rank the 29 Resuscitation Data Items, as we wished to avoid the subjectivity involved in such ranking – we merely attempted to objectively report the presence or absence of these items. Such ranking of these items represents a potential topic of future research.

Despite these limitations, our results should raise awareness about the variation in current neonatal resuscitation documentation across institutions, and should stimulate efforts to generate a core set of essential data items for resuscitation documentation. Ideally, an authoritative body such as the Neonatal Resuscitation Program of the American Academy of Pediatrics or ILCOR should create such a list of core items that will serve as an international standard for neonates, similar to the Utstein guidelines [16] used in adult and pediatric patients.

## Conclusion

Neonatal resuscitation documentation varies significantly amongst different institutions and this variation is attributable to institutional factors. Further institutional efforts at standardizing neonatal resuscitation documentation may further inform neonatal resuscitation research. Our findings could influence national authoritative bodies such as the Neonatal Resuscitation Program to develop a pragmatic, core data set of items for neonatal resuscitation documentation.

## Abbreviations

ILCOR: International Liaison Committee on Resuscitation
NICU: neonatology intensive care unit
NRP: neonatal resuscitation program
VLBW: very low birth weight

## References

[1] Garcia Arias MB, Zuluaga Arias P, Arrabal Teran MC, Arizcun Pineda J (2007). [Factors related to respiratory complications in very low birth weight infants with respiratory distress syndrome]. An Pediatr (Barc).

[2] Kumar P (2006). Physician documentation of neonatal risk assessment for perinatal infections. J Pediatr.

[3] Ma GW, Pooni A, Forbes SS, Eskicioglu C, Pearsall E, Brenneman FD (2013). Quality of inguinal hernia operative reports: room for improvement. Can J Surg.

[4] Kaye W, Mancini ME, Truitt TL (2005). When minutes count--the fallacy of accurate time documentation during in-hospital resuscitation. Resuscitation.

[5] Allan N, Bell D, Pittard A (2011). Resuscitation of the written word: meeting the standard for cardiac arrest documentation. Clin Med.

[6] Smith PC, Araya-Guerra R, Bublitz C, Parnes B, Dickinson LM, Van Vorst R (2005). Missing clinical information during primary care visits. JAMA.

[7] Elder NC, Hickner J (2005). Missing clinical information: the system is down. JAMA.

[8] Berglund S, Norman M (2012). Neonatal resuscitation assessment: documentation and early paging must be improved!. Arch Dis Child Fetal Neonatal Ed.

[9] Niermeyer S, Kattwinkel J, Van Reempts P, Nadkarni V, Phillips B, Zideman D, et al: International Guidelines for Neonatal Resuscitation: An excerpt from the Guidelines 2000 for Cardiopulmonary Resuscitation and Emergency Cardiovascular Care: International Consensus on Science. Contributors and Reviewers for the Neonatal Resuscitation Guidelines. Pediatrics. 2000;106(3):E29. 10.1542/peds.106.3.e29 10969113

[10] Perlman JM, Wyllie J, Kattwinkel J, Atkins DL, Chameides L, Goldsmith JP (2010). Neonatal resuscitation: 2010 International consensus on cardiopulmonary resuscitation and emergency cardiovascular care science with treatment recommendations. Pediatrics.

[11] Kattwinkel J, Perlman JM, Aziz K, Colby C, Fairchild K, Gallagher J (2010). Part 15: neonatal resuscitation: 2010 American Heart Association Guidelines for Cardiopulmonary Resuscitation and Emergency Cardiovascular Care. Circulation.

[12] Hasson F, Keeney S, McKenna H (2000). Research guidelines for the Delphi survey technique. J Adv Nurs.

[13] Harris PA, Taylor R, Thielke R, Payne J, Gonzalez N, Conde JG (2009). Research electronic data capture (REDCap)--a metadata-driven methodology and workflow process for providing translational research informatics support. J Biomed Inform.

[14] Finer NN, Rich W (2002). Neonatal resuscitation: toward improved performance. Resuscitation.

[15] Carbine DN, Finer NN, Knodel E, Rich W (2000). Video recording as a means of evaluating neonatal resuscitation performance. Pediatrics.

[16] Zaritsky A, Nadkarni V, Hazinski MF, Foltin G, Quan L, Wright J (1995). Recommended guidelines for uniform reporting of pediatric advanced life support: the Pediatric Utstein Style. A statement for healthcare professionals from a task force of the American Academy of Pediatrics, the American Heart Association, and the European Resuscitation Council. Resuscitation.

